# Oxidized Xanthan Gum and Chitosan as Natural Adhesives for Cork

**DOI:** 10.3390/polym8070259

**Published:** 2016-07-14

**Authors:** Diana Paiva, Carolina Gonçalves, Isabel Vale, Margarida M. S. M. Bastos, Fernão D. Magalhães

**Affiliations:** LEPABE–Faculdade de Engenharia, Universidade do Porto, Rua Dr. Roberto Frias, 4200-465 Porto, Portugal; dianapaiva@fe.up.pt (D.P.); carol.goncalves8827@gmail.com (C.G.); isavale10@portugalmail.pt (I.V.); mbastos@fe.up.pt (M.M.S.M.B.)

**Keywords:** oxidized xanthan gum, oxidized polysaccharide, chitosan, cork, natural adhesive

## Abstract

Natural cork stopper manufacturing produces a significant amount of cork waste, which is granulated and combined with synthetic glues for use in a wide range of applications. There is a high demand for using biosourced polymers in these composite materials. In this study, xanthan gum (XG) and chitosan (CS) were investigated as possible natural binders for cork. Xanthan gum was oxidized at two different aldehyde contents as a strategy to improve its water resistance. This modification was studied in detail by ^1^H and ^13^C nuclear magnetic resonance (NMR), and the degree of oxidation was determined by the hydroxylamine hydrochloride titration method. The performance of the adhesives was studied by tensile tests and total soluble matter (TSM) determinations. Xanthan gum showed no water resistance, contrary to oxidized xanthan gum and chitosan. It is hypothesized that the good performance of oxidized xanthan gum is due to the reaction of aldehyde groups—formed in the oxidation process—with hydroxyl groups on the cork surface during the high temperature drying. Combining oxidized xanthan gum with chitosan did not yield significant improvements.

## 1. Introduction

Cork (bark of *Quercus suber* L.) is a natural and renewable material with a unique combination of properties, such as elasticity, resilience, impermeability, low density, and very low conductivity of heat, sound and vibration [1]. Most of these properties arise from its distinctive structure. Cork is composed of a prismatic cellular matrix with empty spaces filled with air [2,3]. The main components of cork cell walls are suberin (40% of its dry weight), lignin (20%), polysaccharides (20%) and extractables (15%) [4,5]. The actual composition may vary, depending on the geographic origin, soil and climate, tree age and genetics, interval of harvest, and growth conditions [6]. Cork waste from wine bottle stopper manufacturing is granulated and combined with a polyurethane adhesive to form an agglomerated material with many uses. These include the so-called technical cork stoppers and agglomerated cork panels for insulation or decorative uses. Cork granules can be used in other types of composite materials; for instance, as fillers in epoxy adhesives, allowing for density reduction and impact energy absorption [7,8].

The search for natural materials to be used as green adhesives that are capable of replacing synthetic materials has increased over the years, with consumers demanding high quality products that take into account environmental issues, sustainability, and health concerns [9]. Polysaccharide-based adhesives mostly meet the above criteria, especially for the packaging and food industries [10]. The raw materials are relatively cheap and readily available in nature from vegetable sources or from microorganism production [11]. Low water resistance and poor mechanical performance, however, are common drawbacks of these adhesives.

Xanthan gum (XG)—secreted by the bacterium *Xanthomonas campestris*—is considered an extracellular heteropolysaccharide with a pentasaccharide repeating unit composed of d-glucose, d-mannose, d-glucuronic acid, acetal-linked pyruvic acid, and *O*-acetyl [12]. Xanthan gum has been used in a wide range of contexts, ranging from food to cosmetics and pharmaceutics [13,14]. It can also be combined with other polysaccharides or proteins to produce films, gels, or solutions with different rheological and mechanical properties [15,16,17,18]. Xanthan gum is hydrophilic and water soluble, which limits its use as an adhesive. Use in wood-based particleboards evidenced poor tensile strength and water resistance [19]. Oxidized xanthan gum (XGox) can be obtained through periodate-mediated oxidation, which causes C–C cleavage between (–CHOH)_2_ groups to form dialdehydes. This methodology is commonly used in food industry to introduce a large number of aldehyde groups into polysaccharide chains, improving reactivity [20,21,22]. This not only decreases the gum’s hydrophilicity, but may also provide a route for covalent interaction with the substrate, therefore improving water resistance. Guo, et al. produced films of gelatin with oxidized xanthan gum, showing that the total soluble matter decreased as xanthan gum’s degree of oxidation increased [23]. They observed that the addition of oxidized xanthan gum reduced the gelatin film’s extensibility, probably due to crosslinking induced by the gum’s reactive sites. However, they did not find a relation between the degree of oxidation and the ability to elongate. This was justified by the decrease in the molecular weight of the more oxidized products, which counterbalances the higher rigidity induced by crosslinking.

Chitosan (CS) is obtained from high deacetylation (>50%) of chitin, the second most abundant polysaccharide in nature. It has a linear structure composed of randomly distributed β-(1-4)-linked d-glucosamine and *N*-acetyl-d-glucosamine [24]. The reactivity of the primary amino groups can provide alternative routes for chemical modification. This feature is not available in most polysaccharides, which mainly present hydroxyl groups [25]. The use of chitosan—alone or in combination with other polymers—to form gels, nanoparticles, and films is common in biomedical applications [26,27,28]. Argin-Soysal, et al. produced microcapsules of xanthan gum–chitosan, showing that both constituents can interact efficiently [29]. As an adhesive, chitosan combined with oxidized dextran has been reported to have improved bonding strength, when compared with fibrin glue, for bone or soft tissue gluing applications in surgery [30]. Films of chitosan with oxidized maltodextrin were also reported in the literature for the adhesion of biological tissues. The authors studied the rheological and adhesion properties of the films at different conditions, observing that the adhesive strength increased with increasing concentration of chitosan in the film [24,31]. They also observed that oxidation of maltodextrin was necessary to promote adhesion strength, since films of non-oxidized maltodextrin with chitosan revealed poor adhesion properties. Yamada and co-workers used tyrosinase enzyme to oxidize dopamine and then combined the product with chitosan to improve water resistance of biological adhesives [32]. Chitosan has been used as wood adhesive, both alone or combined with other polymers. Patel, et al. evaluated the adhesive potential of different chitosan-based formulations using a wood double-lap shear test. They observed that chitosan’s bonding performance can be enhanced by adding glycerol and trisodium citrate dehydrate in the adhesive solution, especially with regard to water resistance [33]. Umemura and co-workers applied chitosan and konjac glucomannan as adhesives for plywood and tested the dry and wet bond strengths. They concluded that a combination of both components enhanced the dry strength and significantly improved the water resistance [34].

The viability of using two widely available polysaccharides (XG and CS) as cork binders was studied in the current work. The adhesives were used as aqueous dispersions with concentrations up to 6% (*w*/*w*). Chemical oxidation of XG was studied as a strategy to provide water resistance and reactivity. Neither of these polysaccharides have been previously reported in the literature as an adhesive for cork.

## 2. Materials and Methods

### 2.1. Materials

Xanthan gum (XG) from *Xanthomonas campestris* was purchased from Sigma Aldrich, St. Louis, MO, USA. Chitosan (CS, degree of acetylation of 90%), was acquired from Golden-Shell Pharmaceutical Co. Ltd., Zhejiang, China. Solvents and materials for nuclear magnetic resonance (NMR) analysis were supplied by Cortecnet, Voisins Le Bretonneux, France. All other chemicals were purchased from Sigma Aldrich. The one-component urethane prepolymer adhesive (PUR), used as reference in tensile strength tests, was provided by Amorim & Irmãos, Santa Maria da Feira, Portugal. This type of adhesive is widely used as a binder in cork granule composites. Cork discs were also supplied by Amorim & Irmãos.

### 2.2. Preparation of Oxidized Xanthan Gum

XG was oxidized using sodium metaperiodate according the procedure described by Serrero and co-workers [31]. Briefly, a solution of xanthan gum 6% (*w*/*w*) was prepared and homogenized with mechanical agitation. Sodium metaperiodate (0.18 M) was added, and the reaction lasted 2 h at room temperature. The resulting solution was dialyzed (Molecular weight cut-off (MWCO) of 12–14 kDa) against water under mild agitation for four days. Water volume was 10 times higher than the dialyzed volume and was replaced four times. 

### 2.3. Oxidation Degree Determination

The degree of oxidation was determined by the hydroxylamine hydrochloride titration method described by Zhao, et al. [35], which is defined as the number of oxidized units per 100 elementary units. It was determined by reacting an excess of hydroxylamine hydrochloride with oxidized xanthan gum. This reaction produces hydrochloric acid, which is then titrated with NaOH and related to the aldehyde content in oxidized xanthan gum. The degree of oxidation was calculated according Equation (1), were *V*_NaOH_ is the consumed volume of NaOH at the equivalence point, *C*_NaOH_ is the concentration of NaOH solution (0.1 M), *n*_C=O_ is the possible number of aldehyde groups, *m* is the dry weight of oxidized XG (0.1 g) and *M* is the molecular weight of the repeating unit of XG (934 g/mol). In the case of xanthan gum, eight aldehyde groups can be formed per repeating unit by the cleavage of the C–C bond between adjacent –CHOH groups.

(1) $$\text{Degree of oxidation}\;\left(\%\right)\;=\;\frac{V_{\text{NaOH}}\times C_{\text{NaOH}}}{n_{C=O}\times m/M}\;\times \;100$$

### 2.4. Nuclear Magnetic Resonance (NMR)

^1^H and ^13^C nuclear magnetic resonance (NMR) spectra were recorded on a Bruker Avance III 400 spectrometer (Bruker Optik GmbH, Ettlingen, Germany), operating at frequencies of 400 and 100 MHz, respectively, using D_2_O (Deuterium oxide) as solvent. Chemical shifts were expressed in δ (ppm), relative to the resonance of trimethylsilyl propanoic acid (TMSP) (0.00 ppm) as an internal standard. Acquisitions were performed at three different temperatures: 26, 40, and 55 °C. Unequivocal ^1^H assignments were made using 2D correlation spectroscopy (COSY) experiments with multiple solvent suppression, while ^13^C assignments were made on the basis of heteronuclear single quantum correlation (HSQC) and heteronuclear multiple-bond correlation (HMBC) experiments obtained with sensitivity improvement and adiabatic bilevel decoupling with adiabatic refocusing.

### 2.5. Preparation of Glues 

Xanthan gum was dissolved in water overnight under mechanical agitation until complete dissolution was achieved. Solutions with different mass concentrations (up to 6% *w*/*w* of solids) were prepared. Chitosan solutions were prepared in the same way, but acidified water was used to ensure complete dissolution. Acetic acid was used to lower the solution pH to 4.5. Oxidized xanthan gum solutions were recovered from dialysis at concentrations close to 2% (*w*/*w*). For high mass concentrations, a rotary evaporator system was used to concentrate the solutions.

After preparation of all solutions, xanthan gum or its oxidized derivative were mixed at the 1:1 and 3:1 mass ratio with chitosan, from samples of equal mass concentrations, to form the adhesive solution at the desirable concentration (6% *w*/*w* of solids).

### 2.6. Total Soluble Matter (TSM)

Films of each combination were also made to analyze the percentage of soluble matter. The solutions were placed in perfluoroalkoxy (PFA) dishes (50 mm diameter) and heated to 120 °C for 2 h. The remaining water was evaporated at 40 °C until constant weight was obtained. The resulting films were immersed in 175 mL of water with magnetic stirring for 24 h and then filtered. The recovered residue (*m*_final_) was dried to constant weight and then related to the initial film weight (*m*_initial_) to give the TSM in percentage, through Equation (2).

(2) $$\text{TSM}\;\left(\%\right)\;=\;1\;-\;\frac{m_{\text{final}}}{m_{\text{initial}}}\;\times \;100$$

### 2.7. Tensile Strength Test

The fresh prepared adhesive solutions were applied in a cork smooth surface with superficial area of 2 cm^2^ and glued to an identical surface. A metallic clamp was used to hold both pieces together under light compression. The system was then heated in the oven at 120 °C for 50 min. After two days at room conditions (temperature and humidity were approximately 20 °C and 70%, respectively), the glued specimens were subjected to the tensile strength test to evaluate the resistance of the adhesive. Water resistance tensile strength test was performed after immersion of specimens in water at room temperature for 24 h, followed by 1 h drying at room temperature.

For tensile strength measurement tests, the samples were clamped in the proximity of the glue joint and a pull-off tension (normal to the glue joint) was applied. A Multitest 1-d tensile test bench was equipped with a BFG 1000 dynamometer (Mecmesin, Slinfold, United Kingdom). The tests were performed at room temperature, under a linear displacement velocity of 0.3 m/h. The maximum tensile strength was obtained at the point of rupture.

## 3. Results and Discussion

### 3.1. Xanthan Gum Oxidation

**Figure 1:**
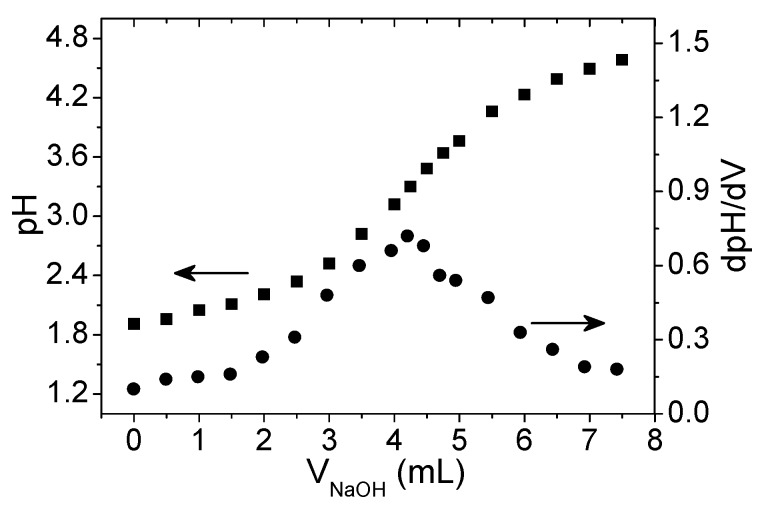
Hydroxylamine hydrochloride titration method for oxidized xanthan gum (XGox) sample with a measured aldehyde content of 61%.

After periodate oxidation and dialysis, the product was subject to hydroxylamine hydrochloride titration to confirm and quantify the aldehyde content. Figure 1 shows a representative example of the pH evolution of the titrated liquid and its first derivative.

The degree of oxidation computed from Equation (1) was 65% ± 8% for 2 h reaction time, based on the measurements from 10 distinct oxidation runs. Serrero and co-workers found similar degrees of oxidation for potato starch under analogous reaction conditions [24,31]. For 24 h reaction time we obtained an aldehyde substitution of 83% ± 3%. This increase in the degree of oxidation with reaction time was expected. However, the more reasonable reaction time of 2 h was adopted for this work. 

**Figure 2:**
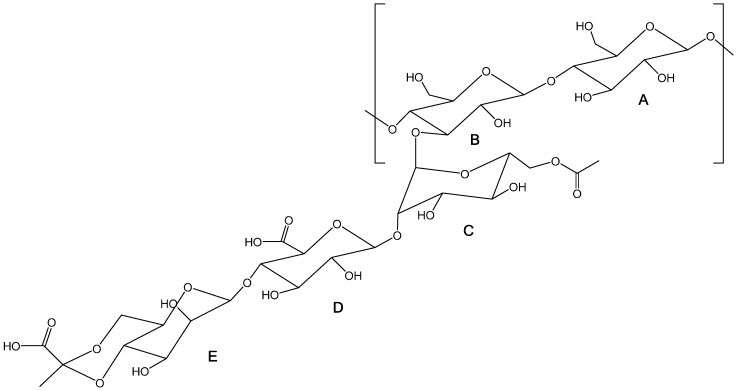
Chemical structure of native xanthan gum molecule: **A**,**B**: β-d-glucose ; **C**: O-acetylated α-d-mannose; **D**: β-d-glucuronic acid; **E**: pyruvated β-d-mannose unit (adapted from [36]).

**Figure 3:**
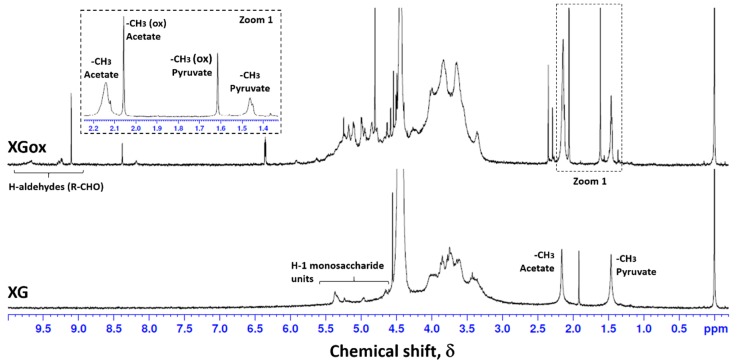
^1^H NMR spectra for xanthan gum (XG) and oxidized xanthan gum (XGox) obtained at 55 °C.

NMR was used to study the modifications occurring in the biopolymer’s primary structure after oxidation. Figure 2 presents the chemical structure of xanthan gum, where letters A to E identify the saccharide units in study. Previous studies on xanthan gum and other polysaccharides were helpful in the interpretation and discussion of the obtained results [36,37,38,39,40,41]. The ^1^H NMR spectra, obtained at 55 °C, for XG and XGox are presented in Figure 3. Table 1 contains the spectroscopic data for the main signal attributions discussed.

**Figure 4:**
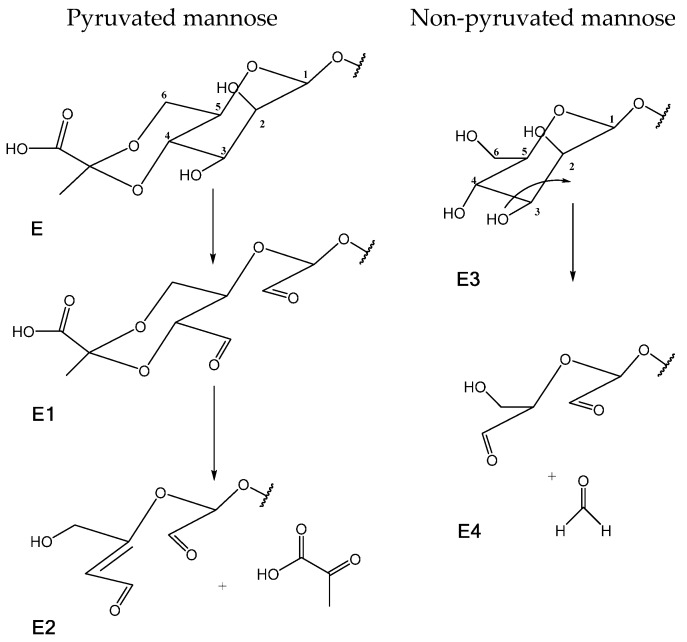
Proposed structures for the terminal mannose unit of the side chain at different stages of oxidation: (**E**) non-oxidized pyruvated mannose; (**E1**) oxidized pyruvated mannose –C_-2_–C_-3_ cleavage; (**E2**) loss of pyruvic acid by β-elimination and formation of a double bond between C_-4_–C_-5_; (**E3**) non-oxidized mannose; (**E4**) oxidized mannose –C_-2_–C_-4_ and loss of C_-3_ as formaldehyde.

In the range between 3 and 4 ppm, it is possible to find most of the chemical shifts of the protons from the saccharide ring, except for the anomeric protons that appear between 4.5 and 5.5 ppm. However, those are difficult to attribute due to peak overlap from the different saccharide units. On the XG spectrum, two distinct peaks from the methyl group of pyruvated (E) (1.458 ppm) and acetated (C) (2.134 ppm) units are visible. At 55 °C, we have quantified the amount of pyruvated and acetated units, since its known that not all units C and E have acetate or pyruvate groups, respectively. Comparing the known amount of internal reference present, we estimated that 43% of C units have an acetate group and 45% of E units have a pyruvate group. This is in accordance with the literature, which indicates the presence of one pyruvate and one acetate group per two polymer side chains [37,42,43]. Other works present different ratios of pyruvate and acetate content, which can be due to the bacterial strain and growth conditions, such as temperature and nutrients present [44,45]. In the XGox spectra, it is possible to note the appearance of two new signals in the same region (1.4–2.5 ppm), attributed to the groups present in the oxidized saccharide units. Interestingly, when comparing the signal for the methyl group for both oxidized and non-oxidized xanthan, it is also apparent that oxidation leads to a loss of pyruvate groups. This shows that oxidation with periodate not only affects the C–C cleavage between adjacent –CHOH groups, but also leads to a loss of terminal groups in the side chain, and therefore results in a polymer with lower molecular weight. Li, et al. also reported that an aggressive oxidation approach can lead to the degradation of the oxidized polysaccharide units, either in acidic or alkaline media [22]. To better understand these phenomena, interpretation of other signals was taken into consideration. The proton NMR spectra also exhibits new signals in the region of 9 ppm, attributed to the presence of aldehyde groups formed during the oxidation process. The chemical shift at 9.103 ppm is attributed to an aldehyde proton in the vicinity of a C=C bond. This interpretation was based on the 2D heteronuclear correlations (HSQC and HMBC) obtained for the oxidized polymer. From the signals obtained, we can propose the formation of new structures at different stages of oxidation (Figure 4).

Structure E2 was predicted following the correlations obtained in HSQC and HMBC mode. The aldehyde group (HC=O, 9.103 ppm/191.210 ppm) was revealed to be in the vicinity of an ethylenic carbon. HMBC data revealed a carbon signal at 152.060 ppm, meaning the presence of a quaternary carbon (qC) that was also neighbouring a carbon (126.686 ppm) linked to a proton with signal at 6.354 ppm. With this information, we were able to predict structure E2, assuming the previous form (E1) of the pyruvated mannose.

Correlating the values obtained for the proton signals at 9.103 ppm (aldehyde) and 6.354 ppm (HC=qC) for the presented structure (E2) with the ones obtained for the methyl group of the non-oxidized (E) and oxidized (E1) pyruvated unit, we are able to say that these three structures represent the total amount of pyruvated saccharide units in the original xanthan gum. In structure E1, the saccharide ring opened between C_-2_ and C_-3_ and formed two aldehyde groups. In some repetitive units, this structure is stable and was confirmed by the shift to lower field of the CH_3_-pyruvated mannose (Figure 3). However, some of the E1 structures suffered further attack due to the slightly acidic conditions. After the formation of the aldehyde groups, a β-elimination reaction could occur, leading to the formation of the third structure (E2) with the release of pyruvic acid, which is then removed during dialysis. Although β-elimination is more likely to happen in alkali conditions [46,47], Veelaert, et al. reached similar results studying physicochemical transitions on oxidized potato starch at acidic pH. They also verified a decrease of the molecular weight of the oxidized polymer, which was attributed to acid hydrolysis that may result in chain scission [48]. This phenomenon was not studied by us, but is expected to occur.

On structure E2, the loss of pyruvic acid was suggested by the lower field shift of 2H_-6_ (4.482 ppm, dd, *J* = 3.6 and 19.2 Hz; 4.598 ppm, dd, *J* = 2.4 and 19.2 Hz) when compared to the respective signals of XG (3.7–4.0 ppm), as well as by the lack of other CH_3_ pyruvated signal. The coupling constants (dd, *J* = 2.4, and 3.6 Hz) of the ethylenic proton at 6.354 ppm (C_-4_) suggest that this proton has the ability to establish an allylic coupling with 2H_-6_.

The low intensity of proton signals in the region of 9–10 ppm can be explained by the fact that such aldehyde groups can be in equilibrium with hemiacetal groups, as proposed by other authors [31,49,50]. Another aspect found in the literature is the behaviour of terminal mannose units, where three hydroxyl groups can be found in subsequent carbons. The oxidation pattern expected for those groups is represented in Figure 4 (E3 and E4 structures). The periodate attack to the mannose unit E3 happens in two locations: between C_-2_–C_-3_ and C_-3_–C_-4_. As suggested in the literature, the double cleavage leads to the release of C_-3_ as formaldehyde, which was later removed by dialysis [51,52,53]. The C_-6_ carbon (60.740 ppm) identified in unit E3 is linked to two protons. Through 2D HMBC correlation, one of those protons (3.654 ppm) is correlated to the aldehyde group (HC=O, 9.223 ppm/194.137 ppm) attributed to the terminal carbonyl group of the non-pyruvated mannose unit at the end of the side chain. The terminal mannoses were more visible, and therefore easily identifiable due to the mobility of the molecules at the end of the side chain. The high molecular weight and complexity of the polymer reduce the internal mobility of relevant chemical groups, and make the identification of other relevant signals present in the oxidized molecule difficult.

### 3.2. Total Soluble Matter

In order to evaluate water resistance, free-standing dry films obtained from different glue formulations were immersed in water for 24 h at room temperature. Table 2 shows the total soluble matter (TSM) results. XG and CS films were completely solubilized during contact with water, yielding a viscous solution. On the other hand, oxidized xanthan gum films were not completely soluble, a direct consequence of the structural changes induced by oxidation. The mixtures of XG with CS at a 1:1 mass ratio show low TSM values. However, the films evidenced swelling after water immersion, increasing in thickness by at least three times and acquiring a gel-like consistency. The mixtures of XGox with CS at a 1:1 mass ratio evidenced the best behavior, yielding the less soluble films and exhibiting no swelling. After the test, the XGox:CS films remained resistant to handling. 

### 3.3. Tensile Strength

**Figure 5:**
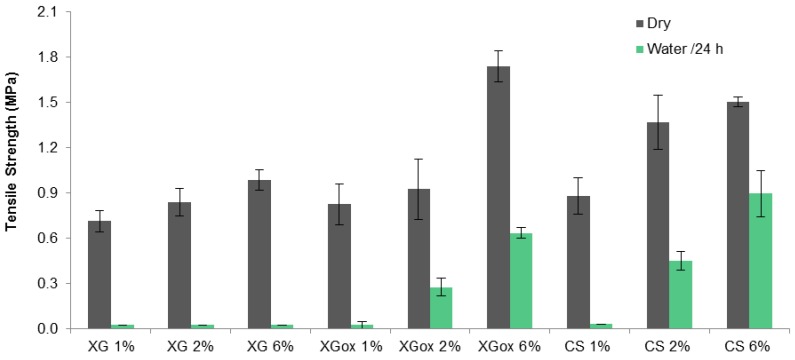
Tensile strength and water resistance of cork joints glued with xanthan gum, oxidized xanthan gum, and chitosan adhesives for different concentrations. Error bars represent standard deviations for a minimum of five measurements.

The tensile strength results obtained for the glued cork samples with different adhesives at different mass concentrations in solution are shown in Figure 5. In all cases, there is a tendency for improving strength with increasing concentration. After immersion in water for 24 h, joints glued with xanthan gum (XG) exhibit null bond strength for all concentrations tested. Oxidized xanthan gum (XGox), on the other hand, shows significantly better water resistance, in agreement with the TSM results, especially at the highest concentration. Interestingly, XGox at 6% yielded the best tensile strength result of all glues. The aldehydes present in the oxidized gum are probably able to react with hydroxyls in the cork structure, and to form a crosslinked adhesive structure upon heating. Chitosan glue (CS) exhibits a behavior similar to XGox, but with slightly higher water resistance. Chitosan’s known adhesive performance can be attributed to two main factors. On one hand, it is able to establish electrostatic interactions, hydrogen bonding, and van der Waals forces between d-glucosamine and the adherend. On the other hand, it has good wetting capability, due to low surface tension and a high dispersive component of the surface energy [54].

**Figure 6:**
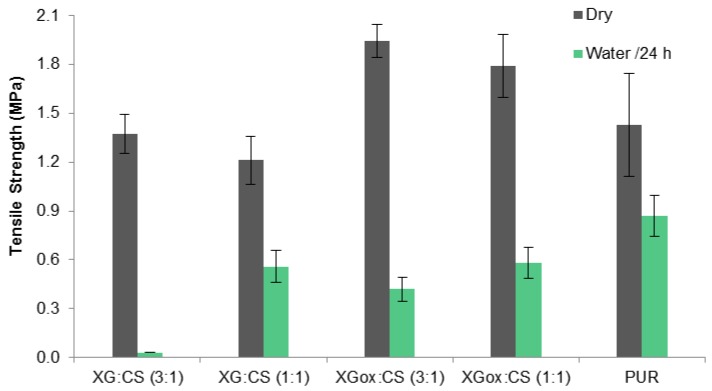
Tensile strength and water resistance of cork joints glued with xanthan gum–chitosan and oxidized xanthan gum–chitosan glues (6% concentration). Error bars represent standard deviations for a minimum of five measurements.

XG, XGox, and CS were combined in different ratios in order to evaluate the possibility of a synergistic effect resulting from physico/chemical interaction between the two. Figure 6 represents the tensile strength and water resistance of mixtures for two different gum:chitosan mass ratios. The results are compared to the synthetic polyurethane adhesive traditionally used for bonding cork.

Mixtures of XG and CS show worse performance than CS alone, indicating poor interaction between chitosan and xanthan gum. The combination of XGox and CS leads to better results, but still below the performance of CS alone in terms of water resistance.

In the tensile strength tests, chitosan and the synthetic adhesive show similar behavior. Since CS shows total solubility as a standalone film, the good water resistance of the glued joint is probably associated with interactions with reactive groups present on the cork surface, such as addition to epoxide ring. Oxidized xanthan gum proved to be a good adhesive for cork. Even though we could not obtain direct evidence of this, it is expected that aldehyde groups present in XGox would react with hydroxyls on the cork cell walls, forming hemi-acetals. As with chitosan, this interaction may justify why XGox films show poor water resistance (TSM test), but achieve very good results in the tensile strength tests of glued joints after immersion in water. Combination of oxidized xanthan gum with chitosan had the potential to improve the adhesion properties due to crosslinking of the aldehydes with the amino groups to form an imine linkage (Schiff base). This reaction, however, competes with the interaction of aldehydes with cork surface, and the net result is therefore uncertain. In this work, we observed no improvement in performance for this mixture.

## 4. Conclusions

Xanthan gum was successfully oxidized with periodate. The aldehyde content after oxidation, determined by hydroxylamine hydrochloride titration, was 65% ± 8%. NMR was used to characterize the oxidized polymer. New structures have been identified as a result of the oxidation process, which mainly attacked the side chain of the polymer, probably due to the higher accessibility of those saccharide units.

Cork joints glued with xanthan gum showed no water resistance. Oxidized xanthan gum, on the other hand, exhibited water resistance close to that of chitosan and polyurethane adhesives. Interestingly, it showed the highest dry bond strength of all adhesives tested. It is hypothesized that the good performance of oxidized xanthan gum is associated with the reaction of aldehydes formed in the oxidation process with hydroxyls in the cork surface. No significant improvement was observed by combining oxidized xanthan gum with chitosan, when compared to the individual performance of the adhesives.

Both oxidized xanthan gum and chitosan deliver promising performances as natural adhesives for cork, namely in terms of water resistance.

## Figures and Tables

**Table 1:** Relevant spectroscopic data (^1^H and ^13^C NMR) of oxidized xanthan gum in D_2_O.

δ (ppm)		Assignment
^1^H	^13^C	
1.458	27.615	CH_3_ of pyruvated mannose (E)
1.615	26.266	CH_3_ of oxidized pyruvated mannose (E)
2.071	23.605	CH_3_ of oxidized O-acetylated mannose (C)
2.134	23.423	CH_3_ of O-acetylated mannose (C)
3.654	60.740	CH_2_ (C_-6_) of non-pyruvated mannose (oxidized) (E4)
4.482	64.340	CH_2_ (C_-6_) of mannose after β-elimination (E2)
4.598	64.340	CH_2_ (C_-6_) of mannose after β -elimination (E2)
6.354	126.686	CH ethylenic carbon of mannose after β-elimination (E2)
–	152.060	qC ethylenic carbon of mannose after β-elimination (E2)
9.103	191.210	HC=O of mannose after β-elimination (E2)
9.223	194.137	HC=O of non-pyruvated mannose (oxidized) (E4)

**Table 2:** Total soluble matter (TSM) and aspect observations of films of xanthan gum (XG), oxidized xanthan gum (XGox), chitosan (CS), and mixtures at 1:1 mass ratio. Standard deviation is for a minimum of three measurements.

Adhesive	TSM (%)	Observations
XG	100	Film dissolves completely, forming a solution with low viscosity.
XGox	78 ± 5	Film divides into smaller pieces and dissolves partially; no swelling observed in the surviving film pieces.
CS	100	Film dissolves completely, forming a highly viscous solution.
XG:CS	31 ± 2	Film dissolves partially, but maintains its integrity; swelling is visible, especially in the borders.
XGox:CS	20 ± 2	Film dissolves partially, but maintains its integrity; no swelling is visible.

## Abbreviations

COSY	Correlation spectroscopy
CS	Chitosan
DA	Degree of acetylation
D_2_O	Deuterium oxide
HSQC	Heteronuclear single quantum correlation
HMBC	Heteronuclear multiple-bond correlation
MWCO	Molecular weight cut-off
NMR	Nuclear magnetic resonance
PFA	Perfluoroalkoxy
PUR	Polyurethane adhesive
TMS	Total soluble matter
TMSP	Trimethylsilyl propanoic acid
XG	Xanthan gum
XGox	Oxidized xanthan gum
